# Supplementary material for: Less is more: Efficient behavioral context recognition using Dissimilarity-Based Query Strategy
Source: PLoS One. 2023 Jun 7;18(6):e0286919. doi: 10.1371/journal.pone.0286919 (PMC10246805; doi:10.1371/journal.pone.0286919)
Supplement: Attachment — Submitted filename: Response to Reviewers.docx [file pone.0286919.s001.docx]

**Dear Dr. Luigi Borzì**

**Academic Editor**

**PLOS ONE**

**We greatly appreciate your time and efforts in handling our manuscript, “Less is more: efficient behavioral context recognition using DBQS”. We are also grateful to the reviewers for their valuable comments. In the revised manuscript we have incorporated all of the reviewers’ comments and we believe this helped substantially improve the manuscript’s quality.**

**Please find below the point-by-point response to the reviewers’ comments. We look forward to hearing from you soon.**

**—--------------------------------------------------------------------------------------------------------------------------**

**Reviewer #1: Dear authors, thanks for your submission. The paper tackles an interesting problem and seems to achieve quite good results, even if based on quite a simple intuition. I think nevertheless that some aspects could be improved:**

**1. all references to Sections are absent (e.g., you write "in Section ", but there is no section number following).**

Thank you for taking the time to respond to this. The paragraph on lines 94 and 95 have been revised.

**2. the concept of active learning is referred many times in the text from the introduction but is not clearly explained until the method section. I think this description should be anticipated in the background for a more coherent and readable introduction**

Thank you for this suggestion. We have introduced the Active Learning concept in the introduction section on lines 72 and onwards. Later on, we are also introducing the Active Learning technical details in the Related Work section on line 205 and onwards.

**3. please check the acronyms, then use the full name just the first time and then only the acronym. For example, the acronym definition for active learning is repeated several times in the text. Consider adding an acronym list**

Thank you. Yes, I have added a list of acronyms at the end of the paper. Our acronym list contains 12 acronyms on page 25

- Active Learning (AL)
- Dissimilarity-Based Query Strategy (DBQS)
- Time Variation (TV)
- Watch Accelerometer (WAcc)
- Phone State (PS)
- Early Fusion (EF)
- Balanced Accuracy (BA)
- Best Performance (BP)
- Comparable Performance (CP)
- Convolutional Neural Network (CNN)
- Human Activity Recognition (HAR)
- Long Short Term Memory (LSTM)

**4. table 1 is never referenced in the text**

Thank you for pointing this out we have refered to Table 1 on Line 255 of the paper

**5. formula 8 has a . instead of a multiplication dot**

Thank you for pointing this out. Equation (8) has been corrected appearing after line 444 of page 12.
**6. summarize numerical results in tables, reporting balanced accuracy values in text especially page 14 is not an effective description**

Thank you for the good suggestions. We have added a table (table 3) on page 15 after line 526 reporting the balance accuracy for all sensors and their annotation load in percentage.

-------------------------------------------------------------------------------------------------------------------------

**Reviewer #2: In this paper, the authors propose a novel behavior context recognition approach i.e., Dissimilarity-Based Query Strategy(DBQS). The approach DBQS leverages Active Learning based selective sampling to find the informative and diverse samples in the sensor data to train the model. The results are corroborative and interesting. I can recommend this paper for a publication after several main concerns are addressed.**

**1. I recommend the authors to divide Section 1 (introduction) into the following subsections: Background, Limits of Prior ATRs, Research motivation, Main contribution.**

Thank you for the suggestions. Section Introduction from lines 1 to 95 (pages 1 - 3) has been divided into the following subsections

- Background: Containing the background of the domain
- Limitations of prior research: Limitations of the currents literature in the domain
- Research motivation: Describes the motivation behind the work
- Main contribution: Lists the contribution of our proposed approach

**2. How do the window length and overlap influence the final performance? The configuration details about each benchmark dataset should be introduced.**

Thank you for this valuable suggestion and we agree. We acknowledge the significance of different window lengths in the recognition process. For this, we have also executed the experiment with a window length of 40 seconds for the proposed methodology to analyze the impact on the final performance. For analysis against all sensor data, we have added graphs on page 16 (Fig 4) and also discussed the results on page 16, paragraph starting from lines 556--559.

**3. The confusion matrices should be added.**

Thank you for the suggestion. We have rerun the experiment with 80% and 20% training and testing sets, respectively, and drew the confusion matrix for each sensor separately using matplotlib (python library). Active Learning is an iterative process, so we have extracted the confusion matrix where the balanced accuracy stabilizes. We have added Fig. 6, 7, and 8 for confusion matrix at the end of page 16 and on the top of page 17. Further, its details are given on page 16 with a paragraph starting from lines 570-582.

**4. In fact, there has been high imbalance in activity benchmark datasets. It will be better if the authors could analyze or discuss how the active learning help to alleviate this issue.**

Thank you for this suggestion. We have discussed the imbalanced class problem of the HAR dataset with its impact on recognition performance on page 18, lines 572-582. Further, we have discussed in detail how the proposed method deal with the imbalance class problem using the clustering approach. During the Active Learning process, the clustering approach maintains the same class ratio of the whole training data in each successive batch. See the section Discussion paragraph starting from lines 640 to 649 on page 18.

**5. I also recommend the authors to refer to several recent activity recognition literatures in introduction part.
a. 10.1109/TETCI.2021.3136642
b.10.1109/TIE.2022.3161812
c. 10.1109/TMC.2022.3174816
d. 10.1145/3551486
e. 10.1109/TIM.2021.3102735**

Thank you for the recommendation. I have added all the mentioned articles in the introduction section.

10.1109/TETCI.2021.3136642 (reference [10], page 1 on line 9)
10.1109/TIE.2022.3161812 (reference [20], page 2 on line 31)
10.1109/TMC.2022.3174816 (reference [18], page 2 on line 30)
10.1145/3551486 (reference[15], page 2 on line 30)
10.1109/TIM.2021.3102735 (reference[16], page 2 on line 30)
