# Supplementary material for: Less is more: Efficient behavioral context recognition using Dissimilarity-Based Query Strategy
Source: PLoS One. 2023 Jun 7;18(6):e0286919. doi: 10.1371/journal.pone.0286919 (PMC10246805; doi:10.1371/journal.pone.0286919)
Supplement: Attachment — Submitted filename: Response to Reviewers (1).docx [file pone.0286919.s002.docx]

**Dear Dr. Luigi Borzì**

**Academic Editor**

**PLOS ONE**

**We greatly appreciate your precious time, efforts and support in handling our manuscript, “Less is more: efficient behavioral context recognition using Dissimilarity-Based Query Strategy”. In the revised manuscript we have incorporated all of the editor’s comments, which significantly raised the quality of the manuscript.**

**Please find below the point-by-point response to the editor’s comments. We look forward to hearing from you soon.**

1. **Please define acronyms the first time they are used. Then use the acronym. Human activity recognition is defined on Line 101 and it is used in full on Lines 135,277; DBQS is defined in the abstract, then it is redefined on**

**Line 659 and used in full on Line 496; Active learning is defined on Line 76 and used in full on Line 76.**

Thank you for pointing this out. We have corrected all the short acronym forms where needed i.e., HAR has been updated on lines 133, 275. DBQS updated on lines 493,656. Similarly, Active Learning has been corrected on line 75.

2. **In the title, I suggest using the full form of DBQS (Dissimilarity-Based Query Strategy) instead of the acronym.**

Thank you for the suggestion. We have updated the paper title to “Less is more: efficient behavioral context recognition using Dissimilarity-Based Query Strategy”

3. **If acronyms are used in tables or images, please define them in the caption (Table 1, Table 3).**

Thank you for pointing this out. We have updated all mentioned tables’ captions i.e., Tables (Table 1 and Table 3) and figures ( Figure 3, FIgure 4, Figure 5, Figure 6 and Figure 8) captions.

4. **References should be ordered (in Line 64 they are not).**

Thank you. We have made all the references in ordered on line 63.

5. **Please review the manuscript and check for typos (e.g. "visulaize" in Line 571).**

Thank you for pointing this out. Corrected the word visulaize to visualize on line 568, word ubiqioutus to ubiquitous on line 46, word natual to natural on line 51, word unlabaled to unlabeled on line 73 and 75, word hybridising to hybridizing

on line 168, word minimising to minimizing on line 178, word labaled to labeled on line 203, word tranined to trained on line 203, word unlabaled to unlabeled

on line 204, word labelling to labeling on line 220 and 221, multi-sensors to multi-sensor on line 278, word senors to sensors on line 286, word sub-sections to subsections on line 296, word evauation to evaluation on line 472, word stabilises to stabilizes on 531, word stabilizies to stabilizes online 533, word analyse to analyze on line 555, word recogniton to recognition on line 556, word clealry to clearly on line 556, word lables to labels on 570

6. **One reviewer suggested some references to their previous published articles. However, this is not fair. Please ignore insignificant references and keep only the necessary citations (e.g. reference 20 proposes a CNN, not**

**an LSTM in Line 31).**

Thank you for the suggestion. We have removed reference 20 from the introduction section on line 30.

7. **The introduction includes only a few sentences. I suggest merging the Introduction and Background sections into one (Introduction).**

Thank you for this suggestion. We have merged the mentioned heading into the introduction, see line 1.

8. **Improve the quality of the figures and ensure that labels and legends are visible.**

Thank you for the suggestion to improve the quality of figures. We have addressed all the issues to the best of our ability. In case, there is any further issue, feel free to share any feedback.
